# ConcentrateNet: Multi-Scale Object Detection Model for Advanced Driving Assistance System Using Real-Time Distant Region Locating Technique

**DOI:** 10.3390/s22197371

**Published:** 2022-09-28

**Authors:** Bo-Xun Wu, Vinay M. Shivanna, Hsiang-Hsuan Hung, Jiun-In Guo

**Affiliations:** 1 Department of Electronics Engineering, Institute of Electronics, National Yang Ming Chiao Tung University (NYCU), Hsinchu 30010, Taiwan; 2 Chunghua Telecom Co., Ltd., Taoyuan 32661, Taiwan; 3 Wistron-NCTU Embedded Artificial Intelligence Research Center, National Yang Ming Chiao Tung University, Hsinchu 30010, Taiwan; 4 Pervasive Artificial Intelligence Research (PAIR) Labs, National Yang Ming Chiao Tung University, Hsinchu 30010, Taiwan

**Keywords:** object detection, Advanced Driver Assistance Systems (ADAS), deep learning, neural networks, embedded system

## Abstract

This paper proposes a deep learning based object detection method to locate a distant region in an image in real-time. It concentrates on distant objects from a vehicular front camcorder perspective, trying to solve one of the common problems in Advanced Driver Assistance Systems (ADAS) applications, which is, to detect the smaller and faraway objects with the same confidence as those with the bigger and closer objects. This paper presents an efficient multi-scale object detection network, termed as ConcentrateNet to detect a vanishing point and concentrate on the near-distant region. Initially, the object detection model inferencing will produce a larger scale of receptive field detection results and predict a potentially vanishing point location, that is, the farthest location in the frame. Then, the image is cropped near the vanishing point location and processed with the object detection model for second inferencing to obtain distant object detection results. Finally, the two-inferencing results are merged with a specific Non-Maximum Suppression (NMS) method. The proposed network architecture can be employed in most of the object detection models as the proposed model is implemented in some of the state-of-the-art object detection models to check feasibility. Compared with original models using higher resolution input size, ConcentrateNet architecture models use lower resolution input size, with less model complexity, achieving significant precision and recall improvements. Moreover, the proposed ConcentrateNet architecture model is successfully ported onto a low-powered embedded system, NVIDIA Jetson AGX Xavier, suiting the real-time autonomous machines.

## 1. Introduction

With the rapid qualitative and quantitative rise of artificial intelligence and deep learning algorithms, the performance of devices based on computer vision is getting much closer to human eye perception. Thus, the related applications have become popular in recent years. Autonomous driving, otherwise termed, Advanced Driver Assistance Systems (ADAS) uses vision understanding for the majority of the system methods, such as object detection, lane line detection, depth estimation, human pose estimation, pedestrian behavior detection and tracking, front vehicle detection, front vehicle detection and tracking, lane departure warning system (LDWS), and so on. Among these methods, object detection is one of the key technologies in ADAS that is used for the Forward Collision Warning System (FCWS), Rear Collision Warning System (RCWS), Blind Spot Detection (BSD), and more. The ADAS can be seen as a second brain lightening the burden of drivers, aiding them in both long and short-distance drives and preventing any possible accidents caused by fatigued drivers.

The existing FCWS in ADAS can only warn about nearby objects conservatively. However, when driving on a highway at 100 km/h and over high speeds, the distant and/or tiny objects are as important as the near and/or big objects to be detected. A quite large amount of accidents lately are happening due to dodging the static objects on the highway, like broken down cars, crash sites, stray and wild animals crossing roads, various kinds of obstacles, and so on. In such conditions, the object detection technique has to detect distant objects, which are small in the frame of the vehicular front camera. Detecting distant, small objects is usually a critical problem in the field of object detection.

A common solution to detect distant objects is using two cameras, one is with a larger field of view (FOV) for both close objects and objects on the diagonal front; the other one is with a smaller FOV for distant objects. This setting can get a high-resolution image of the distant region in the front; however, if the car driving on a curvy road, the small FOV camera may not capture the right region, which is not on the driving path. Additionally, the current systems can only run on resource-limited embedded platforms. To reach a high enough frame rate for the system to react instantly, the computational cost of each part of the ADAS method has to be reduced and processing speed must be increased, whereas using two cameras not only increases product cost, but also increases algorithmic computation cost.

Another method is using a high-resolution image as a model input. Usually, the images are resized to a lower resolution like 640 × 360, to reduce the computational complexity, but if the images with resolution as high as 1920 × 1080 are used as model inputs, it would increase the model complexity by 7–9 times. In addition, according to the research by B. Singh and L. Davis [1], the convolutional operation is not good at a large variance of scales, and it is best to detect objects on the same scale. Typically, the general practice is to use shallower layers’ output features in the convolution neural networks (CNNs) to detect small objects, while deeper layers’ output features are used to detect large objects. However, the output features of the shallower layers may not contain enough information, and the performance may be worse. Although the Feature Pyramid Network (FPN) [2] architecture can be used to overcome this problem, if the scale variation is too large, the model prediction accuracy is still not as expected.

In this paper, a new system termed ConcentrateNet is developed to efficiently improve small and faraway object detection using the vehicular front camcorder visual angle images, utilizing the rule of perception. Multi-scale images are used as inputs in the proposed method to reduce object scale variations. First, the captured high-resolution frames are down-sampled to a lower resolution frame; it only has to detect relatively larger objects, that is, the nearer objects. For distant objects, a deep learning-based vanishing point detection algorithm is used to find the distant region in front of the car driving forwards. Then, the region from the original high-resolution frame is cropped to Region of Interest (RoI) and object detection is performed. In this way, the model is unnecessary to detect such a wide range of object scales and it enhances the processing speed.

**Figure 1:**
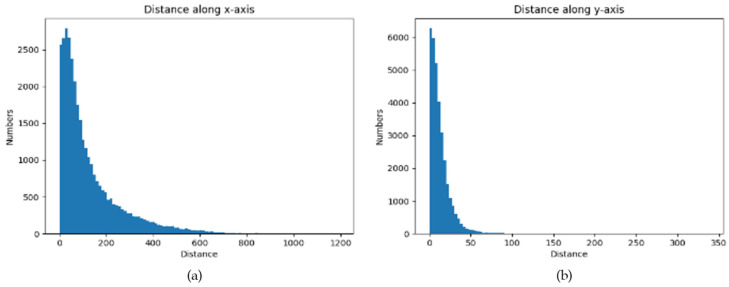
(**a**) Histogram of small objects, (**b**) the vanishing point distance.

To prove that the small objects are near the vanishing point, an analysis of the real front camcorder images dataset, BDD100K [3] is carried out to see how the small objects are distributed in dash cam images. Some post-processing is carried out to perform the labelling in BDD100K and generate the vanishing point location, which is detailed in Section 4, and calculate the distance between the center of small objects, under 32 × 32 pixels, as defined in COCO [4] evaluation metric. The histograms shown in Figure 1 are of the small objects and the vanishing point distance along the *x*-axis and *y*-axis. Using small RoI with 640 × 360 resolution, an acceptable distance range of 320 pixels along the *x*-axis and 180 pixels along the *y*-axis is achieved in the histogram. The ratio of small objects in the acceptable distance range is 90.52% along the *x*-axis and 99.96% along the *y*-axis, indicating that by concentrating on the vanishing point, it is feasible to improve the distant small objects detection in a reasonable way.

## 2. Related Works

V. Růžička and F. Franchetti [5] indicated that object detection benchmarks like Visual Object Class (VOC) and COCO use low-resolution images, but nowadays, image-capturing techniques have been improved so that most of the images are above 1080p or even 4K. If we still use a low-resolution model input size, we will lose the details of small objects in the image. However, using a high-resolution model input size is wasting computation resources. The authors came up with a coarse-to-fine method to reduce computational complexity. First, they do attention evaluation, which roughly detects the whole image by resizing it into a low-resolution model input size. The bounding boxes obtained from the first stage are possibly contained objects, but not sure whether there are still some of the objects overlapping too much. In the second stage, they perform object detection again based on the result from the first stage, cropping the near region of each bounding box. Without down-sampling the image, more details are available. The detection result is more accurate. However, there are some disadvantages associated with this method. Since it is a coarse-to-fine method, if objects are too small that the first stage cannot find the objects, they will not be detected in the end. However, in driving applications, objects usually approach from far to near. Near objects’ locations cannot be used to find distant objects’ locations. Another disadvantage is that the computation is not saved sufficiently if those location distributions of objects are scattered, making the second stage filled with too much of active crops. The method proposed in this paper only needs one larger model input size for near objects and one small model input size for distant objects. Additionally, with the vanishing point detections, we can directly know the small object appearing region, without guessing based on near objects.

AutoFocus [6] proposed by M. Najibi, B. Singh, and L. Davis extended the idea of Scale Normalization for Image Pyramids (SNIP) [1] and improved the performance significantly. The process flow is slightly similar to the previous paper [5], but the performance is far better. They also used the hierarchical method to detect objects from large to small. The detection model contains two branches, one for object detection and one for Focus Chip Generation, which is post-processed from Focus Pixels, an attention model for finding possible smaller object locations. Focus Pixels ground-truth is a binary map combined with all areas of smaller objects, which is defined as an area between 5 × 5 and 64 × 64 pixels in the model input size. The Focus Chip Generation will generate a proper enclosing clip for those focus pixels. Then, it crops the original input image with the enclosing clip and runs the model a second time. After that, it hierarchically detects objects until there is no positive value in Focus Pixels results. The disadvantages of AutoFocus are similar to the previous paper [5], while with the attention module and some of the training tricks, the accuracy is better.

Slicing Aided Hyper Inference (SAHI) [7] proposed a method that can greatly improve the accuracy of small object detection. It can be employed in any object detection model due to the method only adding pre-processes and post-processes in the object detection flow. It slices the input images in overlapped patches, resizes larger ones, and feeds into the CNNs resulting in higher accuracy. Then they carry out their proposed post-processing method to convert back into original image coordinates after Non-Maximum Suppression (NMS). This method is good for improving the detection accuracy of small objects, but it is not exactly suitable for ADAS applications due to the critical issue of its computational efficiency, as this paper did not consider the computing speed.

Maamar et al. [8] present a method that employs a Deep Neural Network (DNN) and K-means to identify groups of customers with similar electricity consumption patterns to understand different types of normal behavior. This method detects anomalies associated with electricity theft in the Advanced Metering Infrastructure (AMI) system, which is a way of detecting the theft by analyzing the billing patterns.

S. Zhou [9] proposed an enhanced SSD method using interactive multi-scale attention features (MA-SSD) uses the attention mechanism to generate attention features of multiple scales and adds it to the original detection branch of the SSD method, which effectively enhances the feature representation ability and improves the detection accuracy. In this method, the feature of different detection scales interacts with each other, and all the detection branches have a parallel structure to ensure detection efficiency. This method is evaluated on the PASCAL VOC dataset.

M. Radhakrishnan et al. [10] presents a new Parametric Segmentation Tuning of Canny Edge Detection (PST-CED) model that is based on the comparison of consecutive segmentation outcomes and the selection of one that yields maximum similarity. In this paper, a hybrid contrast stretching approach was employed depending on the Top-hat filter and Gaussian function.

The proposed ConcentrateNet method can efficiently locate the most possible small objects that appear with vanishing point information, and the core accuracy improvement principle is similar, but have better computational efficiency.

The novelty of the proposed ConcentrateNet is that it proposes a new object detection system for front view images, especially suitable for the systems aiding the ADAS scenes. For applications such as object detection, semantic segmentation and such, many researchers have used different methods like feature extraction CNN models like mobilenet, Visual Geometry Group (VGG), etc. and detectors such as Single-Shot Multibox Detector (SSD), Retinanet, You Only Look Once (YOLO) and so on, to transform the information to detection bounding boxes and build an object detection network like mobilenet-SSD, VGG-SSD. The proposed system, ConcentrateNet is similar to those applications, which can directly replace the detection model components and build a new system like RetinaNet-ConcnetrateNet and YOLOv3-ConcentrateNet to help detect small objects in the ADAS applications that is of great importance from a safety point of view.

## 3. The Proposed ConcentrateNet

**Figure 2:**
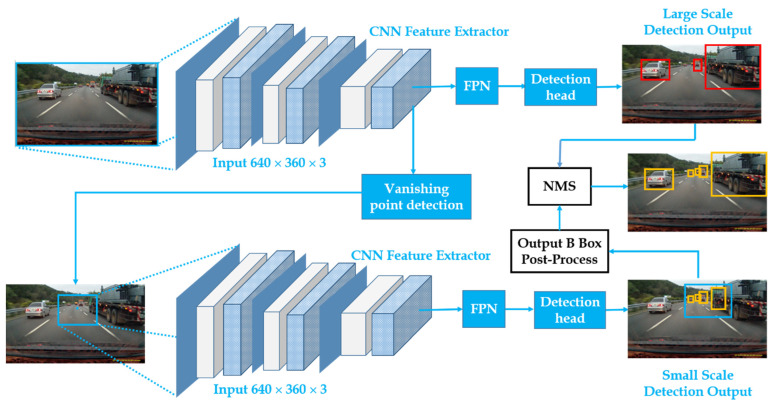
The architecture of the proposed ConcentrateNet model.

The overall process flow of the proposed ConcentraNet object detection model is shown in Figure 2. The first part is the backbone that is used to extract features from the input images. Then there are two subnets viz. object detection subnet and vanishing point detection subnet followed by the post-processing methods for merging large and small RoI results. A detailed discussion of the steps is in the following sections.

On the other hand, in the first step of the inference flow, ConcentrateNet will infer the down-sampled entire image and obtain an RoI bounding boxes and a vanishing point. These bounding boxes are responsible for the detection of large-scale objects irrespective of faraway or near small objects detection. Then, it post-processes the vanishing point into x and y coordinates, which is considered the center of small RoI. In the second stage, with the given RoI size, the detected region is cropped and inferred with the detector without down-sampling. Since there is no down-sampling performed, the image information is the most complete. The generated small RoI bounding boxes are responsible for the detection of distant small objects. In this stage, the vanishing point subnet is not inferred because it is the farthest region. The small RoI bounding boxes add the offset to map back to the original image, and then the big and small RoI results are merged with a post-processing algorithm.

### 3.1. Backbone

In the past, researchers used some rule-based algorithms to extract image features and performed computer vision tasks for object detection such as Scale Invariant Feature Transform (SIFT) [11], Speeded-Up Robust Features (SURF) [12], Histogram of Oriented Gradients (HOG) [13], etc. With the usage of more powerful and efficient Graphics Processing Units (GPUs), parallel algorithms can run significantly faster. With the vast usage of neural networks (NNs) for detection and classification tasks, the NN-based algorithms form the most appropriate kind of algorithm for object detection purposes. A. Krizhevsky et al. [14] are the first ones to utilize a GPU to train a deep NN called Alexnet for a classification task, and achieved a state-of-the-art performance that was far better than the second best in the ImageNet Large-Scale Visual Recognition Challenge (LSVRC)-2010 contest [15]. Since then, a variety of deep NNs are being invented and classic feature extractors are being replaced to be the most used algorithm in various computer vision tasks, and also in speech recognition, semantic recognition, etc. applications.

Some of the state-of-the-art model architectures are GoogleNet [16], Visual Geometry Group (VGG) NNs [17], Residual Neural Network (ResNet) [18], SqueezeNet [19], DenseNet [20], MobileNet [21], EfficientNet [22] and so on. ResNet is one of the most widely used backbone NN. In the proposed design, a ResNet is used as a feature extractor.

In order to train a CNN model to extract image features, and detect and recognize the objects, ImageNet classification dataset is most commonly used. The ImageNet dataset is a huge dataset containing 1000+ classes of objects and more than 10 million images. When training a NN model with such abundant varieties of images, NNs can have a more comprehensive understanding of the object image features like textures, edges, colors, etc.

**Figure 3:**
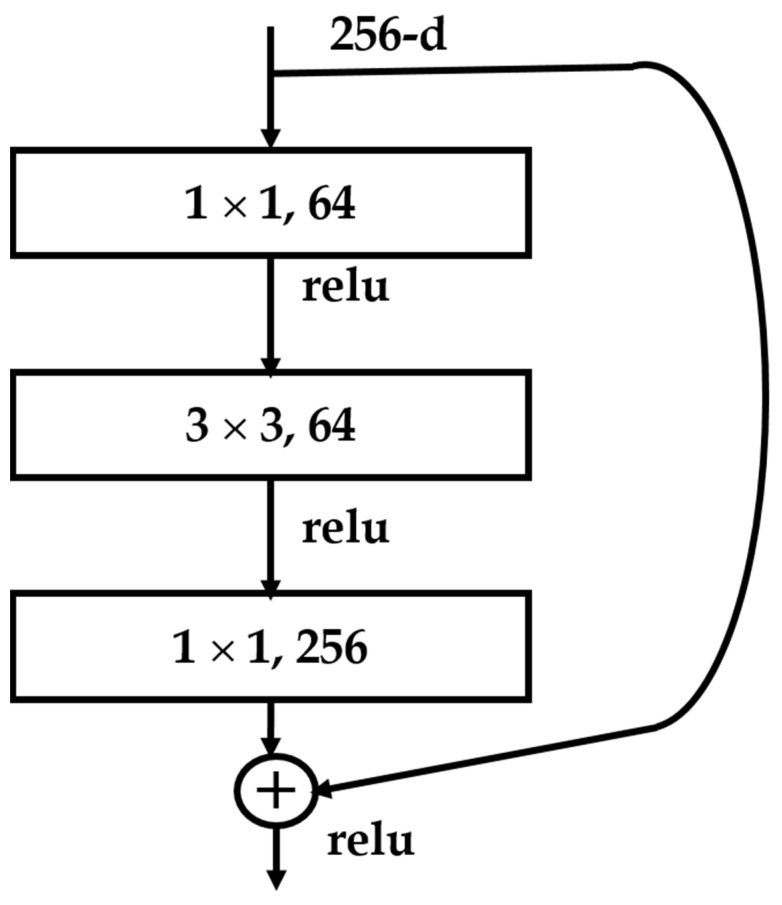
Residual block of Resnet50 [19].

ResNet50 with four levels of feature extraction have different receptive fields to the input images, with the lower level having a narrower receptive field and the higher level having a wider receptive field. Additionally, the shadow layers learn for basic features and the deeper layers learn for complex features. They are composed of a few residual blocks as shown in Figure 3. The last group of layers, the classification subnet, is only used for classification tasks. After training with the ImageNet classification dataset, the classification subnet will be removed, and the rest parts, which are all fully convolutional, are used as an image feature extractor. Table 1 shows the model architecture of the Resnet-50.

### 3.2. Detection Subnet

The detection subnet consists of a neck and dense heads similar to recently proposed many detection models.

#### 3.2.1. Neck

**Figure 4:**
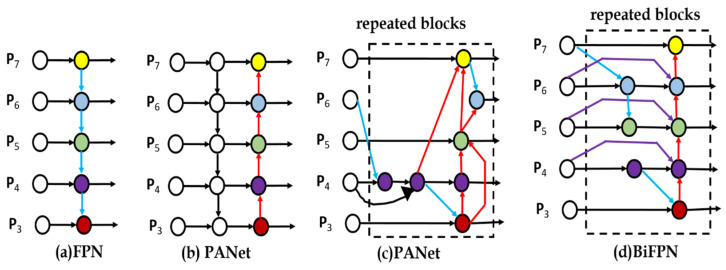
Different necks architecture [22].

The FPN [2] is probably one of the first publications to claim the usage of a neck before the detection of dense heads that can enhance the detection of small objects. A few different kinds of necks such as Path Aggregation Net (PANet) [23], Neural Architecture Search-Feature Pyramid Network (NAS-FPN) [24], Bi-directional Feature Pyramid Network (BiFPN) [22], and so on shown in Figure 4 are proposed based on the FPN architecture dedicating to enhance the combination of high-level rich features and the low-level large receptive field features. In this paper, most of the experimental models use FPN as the neck as it is well known as a basic neck yielding competitive results with the same baseline.

**Figure 5:**
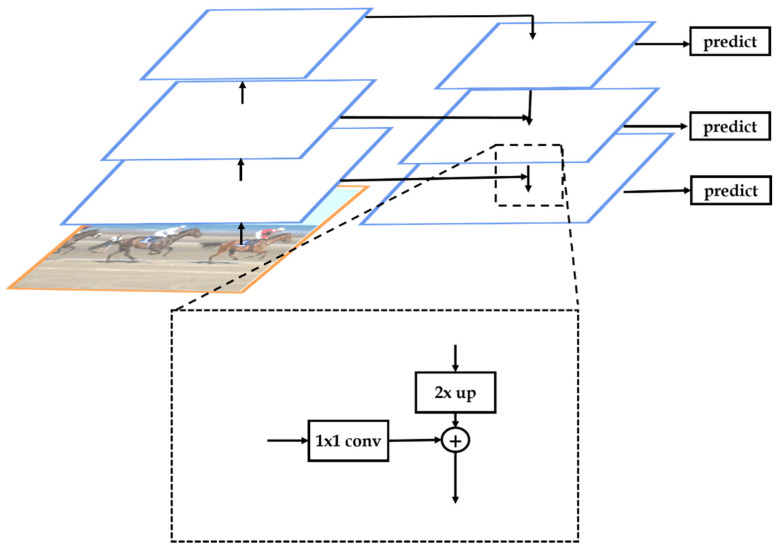
Detailed architecture of FPN [2].

In the detailed architecture of FPN shown in Figure 5, each of the selected feature levels is passed through a 1 × 1 convolutional layer, and the high-level features are up-sampled to their previous level feature share, that is, double the size. Then, these two features are combined forming a new output and it is passed to dense heads.

#### 3.2.2. Dense Heads

The dense heads are the modules responsible for outputting the detected outputs, including the locations of the bounding boxes as well the confidence in its respective detections along with other information based on the task such as the mask information in the instance segmentation task. The detection heads are of two types namely, two-stage and single-stage detection heads. Nowadays, the single-stage models are more accurate and faster compared to the two-stage models. Hence, only single-stage models are used in the proposed method. The bounding box learning methods are also divided into two groups such as anchor-based and anchor-free methods. In the previous state-of-the-art methods, the first version of the YOLO network is anchor-free, having a high inference speed but low accuracy. However, in recent years, more papers are utilizing anchor-free detection dense heads to achieve high accuracies. In this paper, the ConcentrateNet system has been applied to different models of which Fully Convolutional One-Stage (FCOS) Object Detection [25], FoveaBox [26], and Representative Points (RepPoints) [27] are anchor-free models whereas RetinaNet and You Only Look Once v3 (YOLOv3) are anchor-based models.

### 3.3. Vanishing Point Subnet

**Figure 6:**
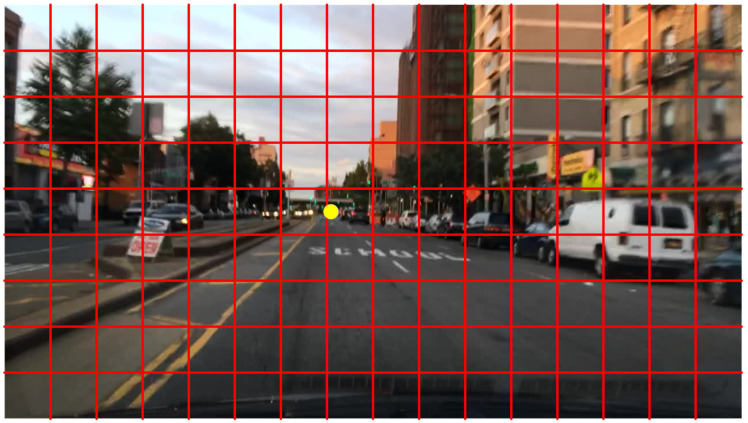
An example of vanishing point grids.

For the proposed ConcentrateNet to concentrate on detecting the traffic at a distance, the vanishing point subnet provides the possible furthest location in the input images. Since the classification process with fewer classes of objects to be detected for ADAS applications, it can be considered the simplest task in deep learning and thus easier to train and achieve high accuracy. As in Deep Learning for Vanishing Point (DeepVP) detection [28], the input image in the proposed work is roughly divided into grids, and the ground truth vanishing point is rounded to one of the grids. Then the classification method is employed to find the vanishing point as in an example shown in Figure 6. In Figure 6, the yellow dot is the vanishing point, and the grid within which it is located in the target of our vanishing point subnet. Although a rough sampling into a grid may cause an inaccurate prediction of an object’s location, it is not key in the proposed system to know the actual vanishing point. The detection of the probable location will suffice.

**Figure 7:**
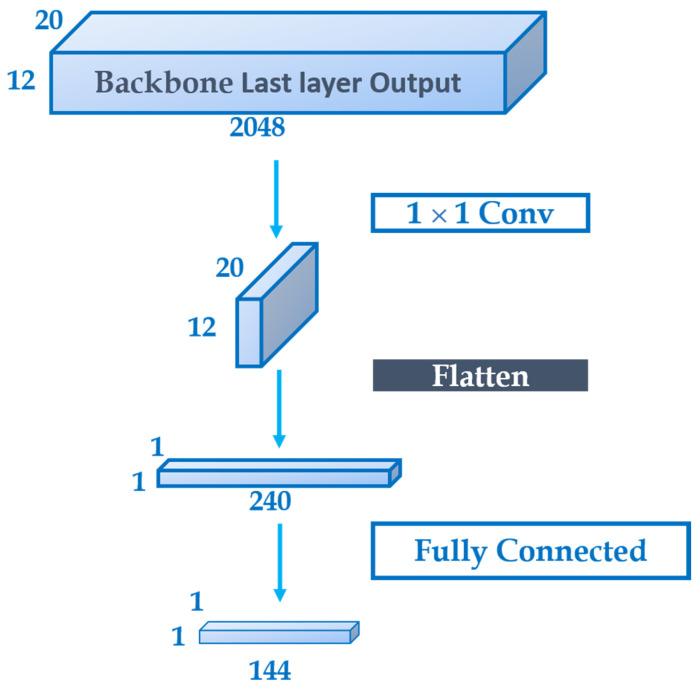
The proposed vanishing point subnet architecture.

The architecture of the vanishing point subnet is shown in Figure 7. A 1 × 1 convolution is used to convert the rich features present in the output of the last layer of the backbone into vanishing point features that are usually remained in only one channel. Then the feature map is flattened into one dimension and connected with a fully connected layer and a softmax layer to carry out the classification of the detected objects.

The architecture of the proposed vanishing point subnet architecture is different from the normal classification subnets that are usually used to average the pooling in order to reduce the dimension into one, instead of using a 1 × 1 convolutional layer followed by a flattening layer. In the experiments of the proposed method, it was tried that using a normal classification subnet proved to be insufficient to train the proposed model successfully, which is due to the average pooling layer dropping too much spatial information crucially required to detect the vanishing points.

DeepVP [28] has used a normal classification subnet to perform the task and yet it was successful in training the model as their whole network is used for training a single task. However, in the proposed method, object detection is the main aim and the weight remaining for detecting the vanishing point are very few. Thus, the proposed vanishing point subnet has to utilize the backbone feature to the best and use the least number of parameters to learn the task of object detection suiting the real-time ADAS applications in contrast to the whole network being used to learn the respective task in DeepVP [28].

Before training, the coordinates of the vanishing point *x* and *y* is to be converted to a class format using the Equation (1).

(1) $$class=y_{VP}\times w_{grids}+x_{VP}$$

where $w_{grids}$ is the number of grids divided along the *x*-axis and *x_vp_* and *y_vp_* are the vanishing points along *x* and *y* axes, respectively. With Figure 3, Figure 4 and Figure 5 as an example, there are 16 × 9 grids, implying $w_{grids}$ is 16 respective to Figure 3, Figure 4 and Figure 5 and the total class is 144.

After inferring the subnet and obtaining the class value, it is converted to a coordinate format using Equation (2).

(2) $$\{\begin{array}{c} x_{VP}=class\;mod\;w_{grids}\times w_{img}÷w_{grids}\;\;\\ y_{VP}=class\;÷\;w_{grids}\times h_{img}÷h_{grids}\;\;\;\;\;\;\; \end{array}$$

where $w_{img}$ and $h_{img}$ denote the width and height of the original image, respectively. The output from the above equations still cannot be used as the center of the small RoI. In order to prevent the small RoI forming out of the image boundary, clamping is required to restrict the center of RoI using $w_{RoI}/2$ to $w_{img}-w_{RoI}/2$ along the *x*-axis and in $h_{RoI}/2$ to $h_{img}-h_{RoI}/2$ along the *y*-axis.

### 3.4. Post-Processing

After the two stages of multi-RoI inference, the ConcentrateNet merges the results of the first stage and the second stage using the bounding boxes merging algorithm shown in Algorithm 1. The output bounding boxes from the dense head in small RoI denoted as {BBoxSmall_n_}, and in big RoI denoted as {BBoxBig_n_}.
**Algorithm 1**: ConcentrateNet multi-RoI Bounding Boxes Merging Algorithm.1:InputBounding Boxes Results in Small RoI $\left\{BBoxSmall_{n}\right\}$, Bounding Boxes Results in Big RoI $\left\{BBoxBig_{n}\right\}$, Vanishing Point Location $\left(x_{VP},\;y_{VP}\right)$, Small RoI Size $\left(w_{RoI},\;h_{RoI}\right)$
2:$\left\{BBoxBig_{k}\right\}=NMS\left(Sort\left({\left\{BBoxBig_{i}\right\}}_{i=0}^{1000}\right)\right)$3:$\left\{BBoxSmall_{l}\right\}=NMS\left(Sort\left({\left\{BBoxSmal_{i}\right\}}_{i=0}^{1000}\right)\right)$4:$w_{offset}=x_{vp}-w_{roi}/2\;$5:$h_{offset}=y_{vp}-h_{roi}/2$6:$\left\{BBoxSmall_{l}\right\}={\left\{BBoxSmall_{i}+\left(w_{offset},h_{offset}\right)\right\}}_{i=0}^{l}$7:$\left\{BBoxSmall_{m}\right\}=RemoveAtEdge\left(\left\{BBoxSmall_{l}\right\}\right)$8:**Output**: $NMS\left(Sort\left(BBoxSmal_{k}\cup^{\;}BBoxSmall_{m}\right)\right)$

The vanishing point obtained from the vanishing point subnet during the post-processing step is denoted as (*x_VP, y_VP*) and the given RoI size is denoted as (*w_RoI, h_RoI*). Initially, NMS is carried out individually with {BBoxBig_n_} and {BBoxSmall_n_} followed by mapping the location of {BBoxSmall_n_} to big RoI, which is the same as that of the input image size, with an addition of an offset. The offset (w_offset, h_offset) is obtained from calculating the left-top coordination of small RoI and adding *w_offset* along *x*-axis and *h_offset* along *y*-axis.

**Figure 8:**
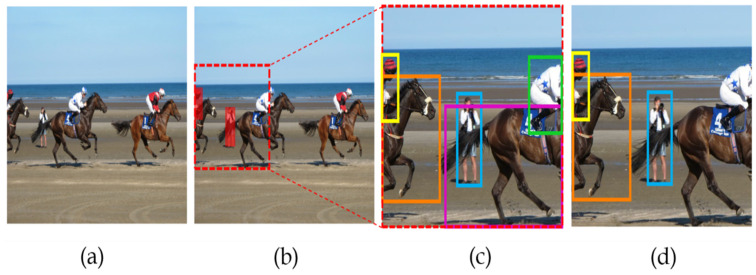
Illustration on bounding boxes removal [27] (**a**) Input image, (**b**) Small RoI, (**c**) Draws all bounding boxes result in the image of small RoI, (**d**) the results after removing at edge bounding boxes.

The vital step in this algorithm is removing the smaller RoI bounding boxes along the edges of the RoI as in AutoFocus [6] and an illustration is shown in Figure 8. The reason for the removal of the bounding boxes at small RoI edges is that those objects may be truncated so that they cannot be eliminated while processing the last part of NMS, merging small and big RoI results. It should be noted that the bounding boxes at the edges of the input images cannot be removed as those objects are also seen as truncated in the big RoI. Therefore, they can be eliminated only if the big RoI is detected successfully.

The final step is to concatenate all the bounding boxes and process NMS to eliminate the duplications of the detected objects in big and small RoI regions. The NMS algorithm used in this paper is Soft-NMS [29], which has proven to enhance the accuracy compared to classic greedy NMS.

## 4. Experiments

The following section discusses experimental details of the proposed method comprising datasets and metrics, the method used in vanishing point generation, evaluation methods, implementation details, and so on. The proposed method is trained and evaluated using BDD100K [3] dataset. Additionally, the proposed method is implemented on a few state-of-the-art object detection networks to evaluate the performance of the proposed method. This section also discusses the method of generating vanishing point labels in the BDD100K dataset, the training detail of the corresponding networks, and the evaluation results compared to the respective original networks. Additionally, the performance efficiency of the ConcentrateNet on embedded systems is also discussed.

### 4.1. Dataset and Metrics

**Figure 9:**
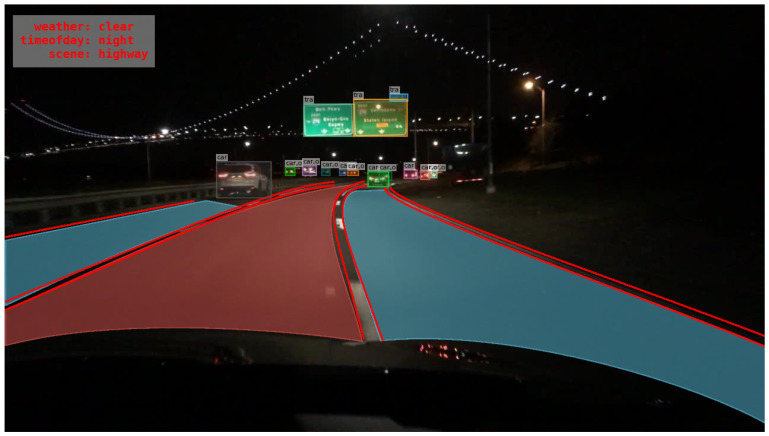
An example of a BDD100K labelling.

BDD100K dataset [3] by Berkeley DeepDrive, contains over a hundred thousand images, with diverse scenes like highways, city streets, tunnels, residential complexes, road intersections etc. The weather and illumination are also diverse, such as clear sky, cloudy, rainy, foggy, day and night conditions and so on. It is suitable for deep learning models to learn the various situations that may be encountered in real-time driving scenarios. The images in the aforementioned dataset are collected from cities in America in contrast to other famous datasets like Cityscapes [30], which comprises traffic environments images from European cities/countries. The BDD100K dataset has labeled object bounding boxes, drivable areas, lane separation lines, and other parameters required for the object detection of autonomous driving applications as in an example shown in Figure 9. The upper left of the image shows the weather, time of day, and scene information, the red lines show the lane separation lines of the road and the red and blue areas of the road indicate the drivable region. There is also information on the object bounding boxes.

### 4.2. Vanishing Point Generation

The vanishing point in an image is generated using the rule of perspective. By extending the opposite lines in the detected image, they will intersect at some point. If the opposite lines are perpendicular to a picture plane, it is considered a vanishing point, which is the farthest location in the direction of the opposite lines. From the driving point of view of the vehicles, the lane lines on the roads are at opposite sites and parallel in the forward direction. To find the farthest location, the vehicle has to be driven forward and the corresponding lane lines are to be processed by extending them and solving to obtain the intersecting point.

**Figure 10:**
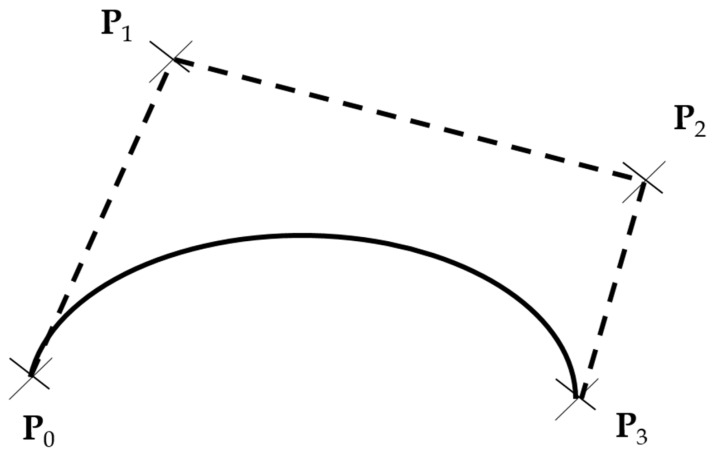
Bezier curves [31].

Before extending the lane separation lines label in the BDD100K dataset, a labeling rule for those lines is to be defined. Berkeley DeepDrive uses the 4th-order Bezier curves and straight lines to represent the lane lines. The 4th-order Bezier curve is a parametric curve that is a function with four points parameters, $P_{0}$ to $P_{3}$ as in Figure 10. There exists an attribute that the tangent line at the end of the curve is equivalent to the connected line of the last two parameter points, $P_{2}$ and $P_{3}$. When extending the lane lines, an assumption is made that the lane lines are not continuously curved, but begin to get straight so that we can extend those lines with tangent lines.

**Figure 11:**
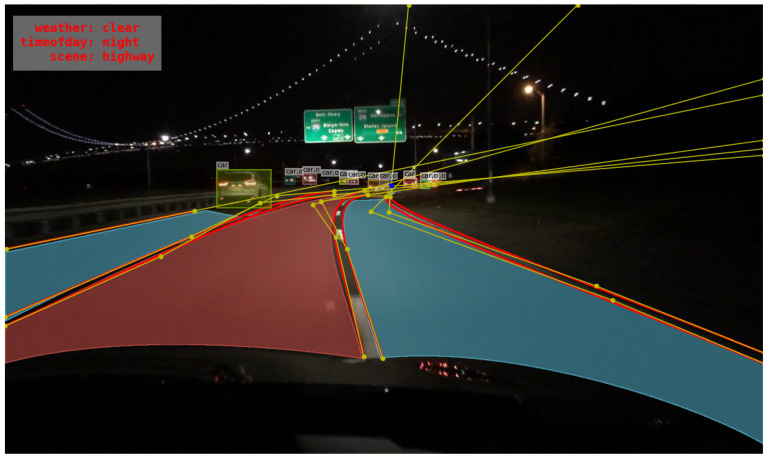
Illustration of lane lines labelling extension.

After loading all the labels of the parallel lane lines, it has to be ensured all the lines’ parameters are labeled starting from the bottom of the image. If not, the inverse of the point list is performed followed by the extension of those lines to the boundary of the image. The illustration is shown in Figure 11 where yellow lines are connected to the Bezier curves parameters and the extended lines. The red lines are the Bezier curves lane lines. Theoretically, the extended lines will intersect at a point if the lane lines are parallel in ideal. However, those lines are manually labeled; they cannot purely use a 4th-order Bezier curve to represent the actual lines and they cannot be parallel either. Due to these non-ideal conditions, the extended lane line usually cannot intersect at one point. Therefore, a linear regression using the least-square method is performed to get one approximate point. If the least-square error is lower than the experimentally set threshold, and the vanishing point is along the boundary of the image, the data can be adopted in the obtained vanishing point dataset. Only the data labeled with more than two parallel lane lines will calculate the vanishing point generation process. The lines perpendicular to the driving main lane are excluded as they may affect the linear regression, which is easy to implement with BDD100K dataset having labeled lane lines of two types, vertical and parallel.

After processing the BDD100K dataset, there are about half of the data satisfy the required conditions. The detailed numbers of data are shown in Table 2. The dataset is large enough to train both the object detection and vanishing point detection task. Note that, the newly released BDD100K detection data in 2020 that has corrected some of the labeling mistakes from the previous version of the dataset is used in the paper.

### 4.3. Object Detection Data Filtering

**Figure 12:**
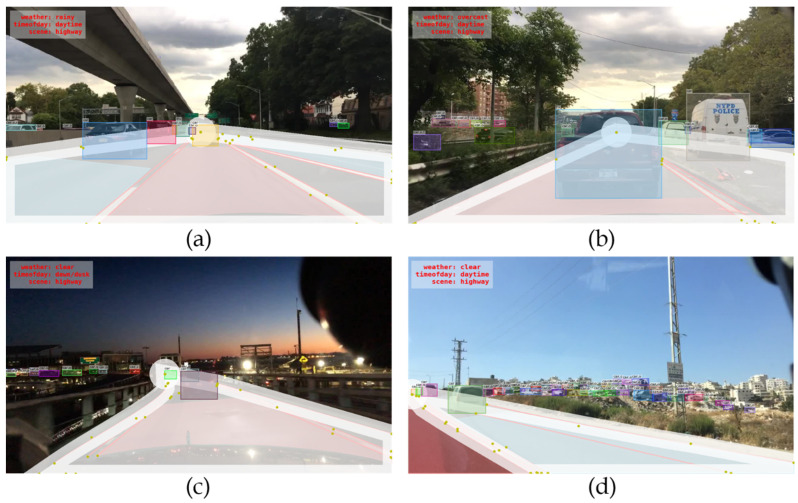
(**a**–**d**) Examples of negligible objects outside the road.

In BDD100K dataset, all the objects that appear in the image are labeled regardless of the location, including the objects that are negligible in size and the possibility to occlude with others is minimal. Some of the examples are shown in Figure 12, in which the road region is labeled with a white mask. To focus on evaluating the accuracy of detecting the objects on roads with which there exists a possibility of a vehicle crashing, the lane markings on both sides of the images are used to enclose an RoI and filter out the ground truth bounding boxes labels that must be detected, apart from these, can be ignored.

**Figure 13:**
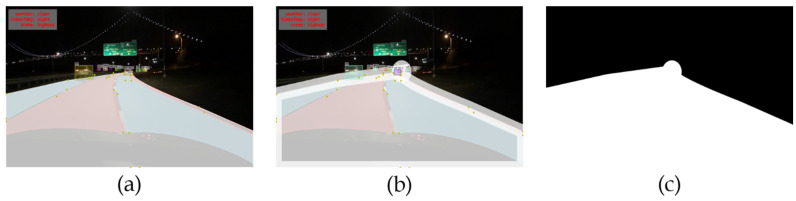
RoI bounding boxes filter generating flow: (**a**) the two sides of lane lines, the vanishing point, and the bottom boundary of the image to enclose a region, (**b**) the RoI is dilated with 50 pixels, and the vanishing point circle is using a radius of 50 pixels, (**c**) the generated bounding boxes RoI mask.

The overall process of filter generating flow is shown in Figure 13. First, the two sides of lane separation lines, the vanishing point, and the bottom boundary of the image to enclose a region are used. Since the region is too close to the edge of the driving road, the objects right beside the road are also considered to be detected in order to ensure safety; a wider RoI is used in this paper. Additionally, a vanishing point cannot fully cover the entire distant region, so a circle to enlarge the vanishing point region is adopted. As shown in Figure 13b, the RoI is dilated with 50 pixels, and the vanishing point circle uses a radius of 50 pixels. Finally, the generated bounding boxes RoI mask is shown in Figure 13c. If the whole bounding box of the object is out of the mask region, such objects are ignored and the object is not counted in evaluation metrics irrespective of it being detected or not.

**Figure 14:**
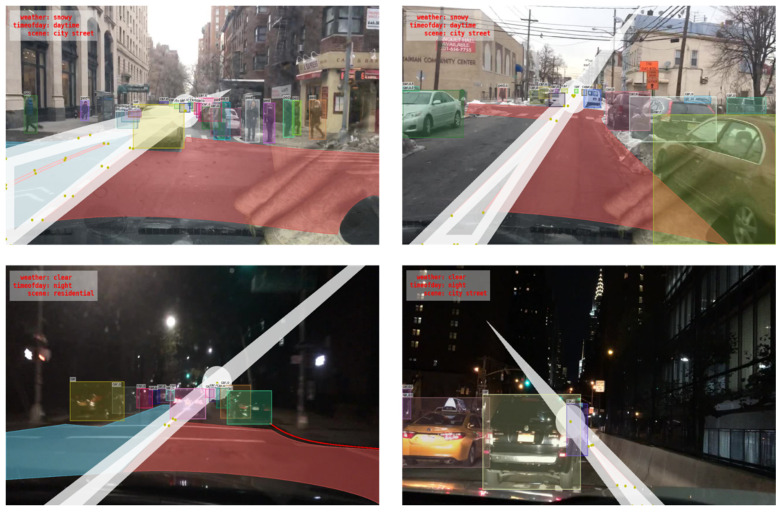
Failed filter mask generation examples.

Using a simple rule-based data processing method may result in some incorrect results. A known problem is that, if a lane marking is occluded by some objects like cars, it will not be labeled. Although using other parallel lane marking is sufficient to generate the vanishing point, it is still not possible to generate the object detection data filter mask because they are not the lane markings on both sides, which make the filter mask incomplete and fails to represent the driving road region. Figure 14 shows a few examples of images that failed to generate a complete filter mask. In such cases, a manual check is performed to validate the dataset and remove the unreasonably labeled data. About 1000 images were removed, and the final number of images used was 5477 as shown in the previous section.

### 4.4. Evaluation Metrics

For object detection purposes, a widely used metric for evaluating quantitative performance is average precision (AP) and average recall (AR). Precision is used to evaluate the ratio of correct predictions to all the predictions, which evaluates the false alarm rate in the result. A recall is used to evaluate the ratio of objects detected successfully in all the ground truth results, which forms the miss rates in the results.

COCO dataset [4] evaluation metrics are adopted in the proposed paper. They introduce detailed metrics and an open-source evaluation tool, which is now the most widely used metric in object detection research. A brief list of the metrics is shown in Table 3. The Intersection of Union (IoU) gives the ratio of predicted results and the ground truth. Higher IoU implies that the prediction location must be more accurate and closer to the ground truth location and shape. Instead of using a specific IoU value for AP, the primary challenge metric calculates the average of AP at 50% IoU, 55% IoU, 60% IoU, and so on up to 95% IoU that can have a wider understanding of the bounding box prediction performance. The AP with a 50% IoU threshold is well known as mean average precision (mAP), which is adopted in PASCAL VOC [32] dataset metrics. There are also metrics of different object sizes. Those sizes are divided into different categories such as small consisting of sizes under 32 × 32 pixels; medium, between 32 × 32 pixels and 96 × 96 pixels; large, above 96 × 96 pixels. These three metrics were tested with an IoU ranging from 50% to 95% followed by a calculation of the average. For the recall, it restricts the total detection number k, which means only the top k confidences of the detection results are collected to calculate the recall because the more the detection bounding boxes, the more the possibility it hits the ground truth. In the metric, this paper uses 100, 300, and 1000 as the top *k* numbers of prediction bounding boxes. In the same way as precision metrics, recall metrics also divide object sizes into small, medium, and large.

For the vanishing point module, the classification task is considered between top1 and top5 accuracies to evaluate the performance. The vanishing point represents the location information, and distance error is used to determine the performance of the proposed method in the application perspective.

### 4.5. Experimental Details and Results

The proposed ConcentrateNet model is implemented on five different object detection architectures namely, the anchor-based RetinaNet [33] and YOLOv3 [34], the anchor-free FCOS [25], FoveaBox [26], and the key-point method RepPoints [27]. With these different implementations of the object detection models, it can be proved that the proposed ConcentrateNet model can be used in most of the recent state-of-the-art detectors and yield a good performance improvement on ADAS applications.

Additionally, the proposed ConcentrateNet can also be applied to most of the single-stage object detection NNs. Except for a few NNs like RetinaNet, YOLOv3, FCOS, FoveaBox, RepPoints, and VarifocalNet on which the ConcentrateNet is tested, a few newer state-of-the-art methods namely, DETR [35], Yolo series like YOLOF [36] can easily extract to a few components such as backbone, neck, bounding box head, and be used in the ConcentrateNet. Thus the proposed method can easily be adapted to new research methods and improve the performance of object detection.

### 4.6. Implementation Details

The proposed ConcentrateNet model is implemented with MMDetection [30], which collects many popular object detection-related modules, including the five models mentioned above. Basically, the default hyper-parameters set in the given configurations are adopted with minor changes in some settings to suit the BDD100K dataset. Due to the configurations set for the COCO dataset, which is much larger than our dataset, the training epoch is changed from default 12 epochs to 48 epochs and the learning rate warmup policy [37] is used at the beginning of training for 1000 iterations with a ratio of 0.33. During the optimization process with the vanishing point module, the gradient explosion often happened. Therefore, the gradient clipping method for optimization is employed. Four Tesla V100 GPUs are used for training, and trained with batch size 64, comprising 16 images per GPU in all the experiments.

For data pre-processing and data augmentation, basic image normalizations with mean and variance in the ImageNet dataset are performed. The images were padded to multiples of 32 due to down-sampling of the backbone five times in total with divisor 2. A multi-scale training with random resizing and random cropping is also adopted and some basic data augmentation methods like random flip and photometric distortions, such as random adjusting brightness, contrast, saturation, and hue are employed. In the above data augmentation methods, random cropping is the most important method to train the vanishing point module, as the vanishing points in the datasets tend to be located at the center of the image, causing the lack of dataset variety. With these data augmentation methods, the performance of vanishing point detection can be improved significantly.

The backbone net used in the paper is ResNet-50, except YOLOv3, which has its own proposed backbone Darknet53. They are all pre-trained with the ImageNet dataset. The neck used is FPN. During training, the bounding boxes classification loss is set to Focal loss [33] as default, bounding boxes regression loss to smooth *L1* loss as default and the vanishing point module uses cross-entropy as default. Since the ConcentrateNet is a multi-task deep learning model, each loss is weighted instead of a direct sum up. The weight of bounding boxes classification loss is 1, bounding boxes regression loss is 2, and the vanishing point module loss is 0.5. In inference, soft-NMS with top 1000 images is carried out, using a confidence threshold of 0.05 and IoU threshold of 0.5 with the parameters not specified.

### 4.7. Vanishing Point Detection Evaluation

The vanishing point detection is a classification task. The *top1* accuracy and *top5* accuracy are used to show the quantitative performance of the proposed method. The evaluation results of different model architectures with a vanishing point module are shown in Table 4.

The input size of the model used is 640 × 360 pixels, with the same model complexity of 0.526 MFlops of the vanishing point module, negligible to the whole ConcentrateNet model complexity. Using the same backbone and same vanishing point module resulted in a few accuracy gaps because of the training loss balancing. To maintain consistency, the same loss weight in each model training is set, but in real applications, to achieve the best vanishing point accuracy, fine-tuning the loss weight parameter for different models is essential. In this paper, the performance improvement with the ConcentrateNet system is achieved instead of training the best performance model for the benchmark. Hence, tuning of these parameters is not performed to retain the consistency of the training parameters.

**Figure 15:**
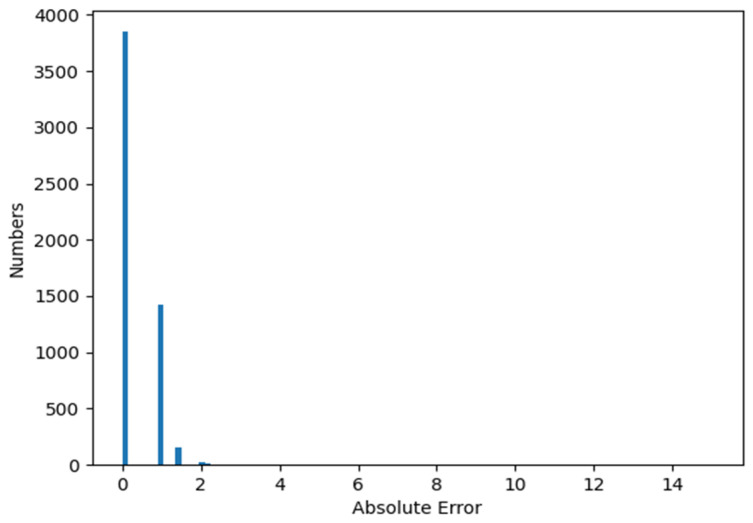
RetinaNet vanishing point error bar chart.

From Table 4, the top1 accuracy might seem not high enough with accuracy ranging from 60% to 70%, but the top5 accuracy is quite high reaching about 98%. The reason is that the ground truth vanishing point labels are generated by rule-based post-processing with the lane line labels, instead of manually labeling, leading to a not accurate enough ground truth. Besides, defining one precisely vanishing point coordinate is not easy. In this paper, the aim is to detect the objects in distant regions rather than the vanishing point, so obtaining higher top5 accuracy is considered satisfactory for the proposed ConcentrateNet. Table 3 shows the average error to the ground truth in the dataset, which is an important metric to evaluate the performance in the application perspective that only has to locate the distant region. The error is obtained by the absolute distance of prediction and ground truth coordinated with the unit grid. It can be noted that there is only about 0.4 grid error on average. Figure 15 shows the bar chart of the error in the validation dataset. The first bar with an error of 0 means the top1 class prediction is the same as that of the ground truth. The second bar with an error 1 indicates the prediction is right beside the ground truth. The third bar with an error of 1.414 means the prediction is on the diagonal side of the ground truth and the other bars with larger errors are in very few proportions. The average error of 0.4 grid is only in a small ratio compared to the small RoI used in the ConcentrateNet, which is used in the experiments with 640 × 360 pixels, that is 8 × 4.5 grids.

### 4.8. Object Detection Evaluation

The five models, the anchor-based RetinaNet [33] and YOLOv3 [34], the anchor-free FCOS [25], FoveaBox [26], and the key-point method RepPoints [27] are trained with the same BDD100K training dataset and the same training hyper-parameters are used to compare with those models trained with ConcentrateNet architecture. The originally proposed models are trained and inferred with input size 960 × 540 pixels, and the proposed ConcentrateNet architecture models are trained and inferred with input size 640 × 360 pixels for both the big and small RoIs. In this condition, the ConcentrateNet architecture models have fewer Flops and slightly more model parameters as shown in Table 5. Even with the less computational cost, the ConcentrateNet architectures are far better than the original architecture for most metrics in the BDD100K validation dataset, as shown in Table 6 and Table 7. The performance of the different object detection models is compared with whether they apply ConcentrateNet respectively and the better metric results are highlighted.

**Figure 16:**
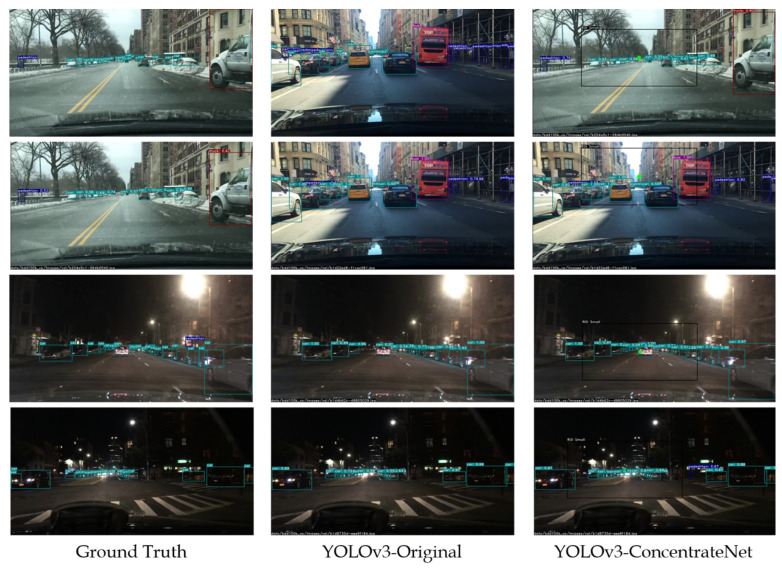
Visualization of model predictions.

Some of the detection results visualizations of YOLOv3 original architecture and ConcentrateNet architecture in different real-time traffic scenarios under various lighting conditions are shown in Figure 16. The left column is the ground truth, the middle column is the bounding box predictions of YOLOv3 original architecture, and the right column is the bounding box and vanishing point prediction of ConcentrateNet architecture, which draws the vanishing point with a yellow circle and small RoI region with a black rectangle. It can be noted that the original architecture misses some of the farthest objects, while with the proposed ConcentrateNet architecture, the model can detect better even in distant regions, which is crucial for the vehicles driven at high speeds to detect the objects at far and still have sufficient time to react and prevent a probable collision.

**Figure 17:**
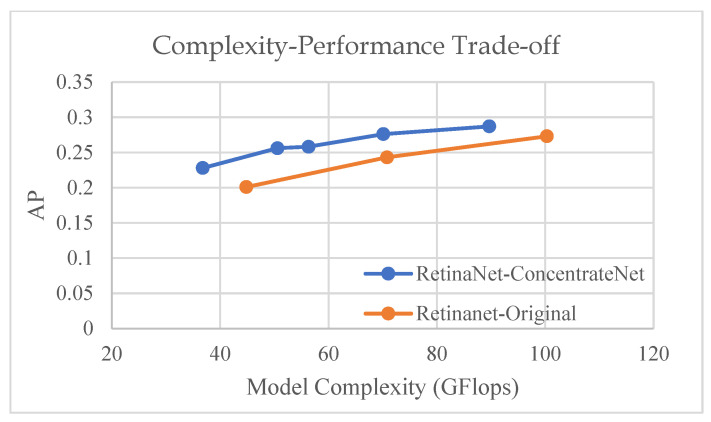
Complexity and performance trade-off comparison.

Different model input sizes are used to evaluate the performance of the proposed method under different model complexity and a comparison graph is plotted, using detection model RetinaNet as an example, as shown in Figure 17. It shows that Retinanet with ConcentrateNet architecture has the best complexity-performance trade-off.

To analyze the model capability, some applications are concerned if the farthest or the smallest of objects that an object detection algorithm can detect. A minimum number of successfully detected objects’ bounding box height or width is counted as shown in Table 8. Note that the bounding boxed output from the models requires resizing back to the original image resolution 1280 × 720, so the width values are of floating type. The original detection method is weaker to detect distant objects with lower resolution. However, with ConcentrateNet method, it is possible to detect the objects at approximately the same distance with an even smaller model input size.

### 4.9. Further Improvement on Detection Model

To compare with the original model performance same as published, we use the same training hyper-parameters and methods like the loss function and post-processing methods. Some of the state-of-the-art methods are experimented in this paper which can further improve the accuracy.

The Soft-NMS post-processing method can easily improve the detection accuracy by changing the suppression way to reduce the confidence level of highly overlapped bounding boxes instead of directly removing them. It can significantly improve the recall performance and a little bit precision performance, as shown in Table 5 Note that we have all applied Soft-NMS to all methods in previous evaluation results.

The Varifocal Loss (VFL) proposed in VarifocalNet [38] is a new object confidence loss function. It learns IoU-aware classification score, merging the objectness confidence and localization accuracy as score. The precision is obviously improved as shown in Table 9.

### 4.10. Implementation on Platforms

The aforementioned methods with ConcentrateNet method were all ported onto the embedded system, NVIDIA Jetson AGX Xavier, developed for AI-powered autonomous machines, with an ARM 64-bit CPU and their own developed GPU for real-time application purposes. The detailed specification is shown in Table 10. Since it uses ARM64 architecture, efforts were put in on building the MMDetection and its dependencies on our own, rather than using the pre-built toolchains available on a free-to-use open source basis, which are usually only built with Intel AMD64 architecture.

During the experimental analysis, the models were implemented on a NVIDIA Tesla V100 and NVIDIA Jetson AGX Xavier and tested for the actual overall inference time as shown in Table 11 and Table 12. The post-process time includes the dense head processing of the bounding boxes’ location and confidence, which also costs time. The inference time of these models is close to each other. Due to the computation parallelized ratio difference and device memory bandwidth limits, the required speed may not be completely achieved due to the given model complexity. We may further increase the parallelism in future work.

## 5. Conclusions

In this paper, a network architecture called ConcentrateNet is proposed to improve the distant objects detection performance, while preserving the near objects detection performance. The proposed ConcentrateNet model performs inference twice, the first time to infer with the whole image, obtaining the big RoI detection results and vanishing point followed by the second time inference with the small RoI by processing the vanishing point information. Then it merges these results with the post-processing algorithm. In this paper, the vanishing point training dataset is built and processed using the BDD100K dataset and manually checked for correctness. From the implementation of the proposed ConcentrateNet architecture, it can be noted that the model possesses less model complexity and achieves significantly higher accuracy. The proposed method is also ported onto different models and successfully implemented on an embedded system NVIDIA Jetson AGX Xavier to suit real-time applications.

## Figures and Tables

**Table 1:** Resnet50 model architecture for ImageNet.

Group of Layers	Layer Composition
Initial downsample	7 × 7 Conv stride 2 + 3 × 3 Max pooling stride 2
Level 1 feature extraction	Residual block ×3
Level 2 feature extraction	Residual block ×4
Level 3 feature extraction	Residual block ×6
Level 4 feature extraction	Residual block ×3
Classification subnet	Average pooling + 1000 node FC + Softmax

**Table 2:** Number of BDD1000K Data.

Title 1	Train	Validation
BDD100K original	69,853	10,000
BDD100K with vanishing points	41,995	5477

**Table 3:** COCO Evaluation Metrics.

Metric Name	Brief Introduction
AP	AP at IoU = 0.5:0.05:0.95 (primary challenge metric)
AP50	AP at IoU = 0.5
AP75	AP at IoU = 0.75
APsmall	AP for small objects: area < 322
APmedium	AP for medium objects: 322 < area < 962
APlarge	AP for large objects: area > 962
AR100	AR given 100 detections per image
AR300	AR given 300 detections per image
AR1000	AR given 1000 detections per image
ARsmall	AR for small objects: area < 322
ARmedium	AR for medium objects: 322 < area < 962
ARlarge	AR for large objects: area > 962

**Table 4:** Vanishing Point Detection Accuracy.

Model Architecture	Top1 Accuracy	Top5 Accuracy	Average Error
RetinaNet	70.26%	98.39%	0.31 grid
YOLOv3	71.66%	98.01%	0.34 grid
FCOS	61.80%	97.43%	0.45 grid
FoveaBox	63.50%	97.88%	0.41 grid
RepPoints	54.57%	94.65%	0.57 grid

**Table 5:** Comparison in model complexity and parameters.

Model Architecture	Input Size	Flops	Parameters
**RetinaNet-Original**	960 × 540	100.32 G	30.92 M
**RetinaNet-ConcentrateNet**	640 × 360 + 640 × 360	89.68 G	30.96 M
**YOLOv3-Original**	896 × 512	86.90 G	61.56 M
**YOLOv3-ConcentrateNet**	608 × 320 + 608 × 320	73.71 G	61.58 M
**FCOS-Original**	960 × 540	95.61 G	29.64 M
**FCOS-ConcentrateNet**	640 × 360 + 640 × 360	85.50 G	29.68 M
**FoveaBox-Original**	960 × 540	98.11 G	30.72 M
**FoveaBox-ConcentrateNet**	640 × 360 + 640 × 360	87.71 G	30.75 M
**RepPoints-Original**	960 × 540	91.54 G	31.29 M
**RepPoints-ConcentrateNet**	640 × 360 + 640 × 360	81.87 G	31.33 M

**Table 6:** Object detection precision evaluation results.

Model Architecture	AP	AP_50_	AP_75_	AP_S_	AP_M_	AP_L_
**RetinaNet-Original**	27.2%	46.8%	26.7%	5.2%	33.2%	56.7%
**RetinaNet-ConcentrateNet**	**28.7%**	**49.1%**	**28.4%**	**8.9%**	**34.3%**	**55.9%**
**YOLOv3-Original**	28.2%	50.9%	26.7%	9.7%	32.0%	54.3%
**YOLOv3-ConcentrateNet**	**30.7%**	**56.4%**	**29.1%**	**11.7%**	**36.1%**	**56.1%**
**FCOS-Original**	22.6%	40.8%	21.3%	6.1%	25.0%	49.1%
**FCOS-ConcentrateNet**	**25.7%**	**46.1%**	**24.6%**	**8.6%**	**28.8%**	**51.1%**
**FoveaBox-Original**	24.9%	44.6%	23.9%	6.8%	29.0%	50.8%
**FoveaBox-ConcentrateNet**	**29.2%**	**51.6%**	**28.2%**	**10.3%**	**33.8%**	**54.6%**
**RepPoints-Original**	24.8%	45.1%	22.8%	6.6%	28.8%	51.4%
**RepPoints-ConcentrateNet**	**25.8%**	**46.0%**	**24.8%**	**8.9%**	**29.0%**	51.4%

**Table 7:** Object detection recall evaluation results.

Model Architecture	AR_100_	AR_300_	AR_1000_	AR_S_	AR_M_	AR_L_
**RetinaNet-Original**	44.6%	44.7%	44.7%	20.5%	55.6%	73.2%
**RetinaNet-ConcentrateNet**	**51.9%**	**53.6%**	**54.0%**	**36.4%**	**61.9%**	**76.4%**
**YOLOv3-Original**	44.9%	44.9%	44.9%	24.9%	52.2%	**70.8%**
**YOLOv3-ConcentrateNet**	**51.3%**	**51.5%**	**51.5%**	**35.7%**	**58.0%**	70.4%
**FCOS-Original**	46.1%	46.7%	46.7%	24.5%	**56.7%**	**73.3%**
**FCOS-ConcentrateNet**	**47.2%**	**48.2%**	**48.2%**	**29.3%**	56.3%	72.6%
**FoveaBox-Original**	44.6%	44.7%	44.7%	22.6%	54.4%	72.0%
**FoveaBox-ConcentrateNet**	**50.1%**	**50.6%**	**50.6%**	**32.1%**	**59.1%**	**73.3%**
**RepPoints-Original**	47.1%	48.0%	48.0%	26.5%	**57.4%**	**73.9%**
**RepPoints-ConcentrateNet**	**48.3%**	**49.8%**	**50.0%**	**33.1%**	56.7%	72.6%

**Table 8:** The minimum width and AP under different input size.

Model	Input Size	Flops	AP	Min Width
Original	960 × 540	100.32 G	27.3	7.47
	800 × 450	70.82 G	24.3	8.68
	640 × 360	44.84 G	20.1	10.01
Proposed Method	640 × 360 + 640 × 360	89.68 G	28.7	6.44
	640 × 360 + 480 × 270	70.13 G	27.6	6.33
	640 × 360 + 320 × 180	56.32 G	25.8	6.13
	480 × 270 + 480 × 270	50.59 G	25.6	6.48
	480 × 270 + 320 × 180	36.78 G	22.8	6.13

**Table 9:** Further Improved Methods Evaluation Results.

Model	AP	AP_50_	AP_75_	AP_S_	AP_M_	AP_L_	AR_100_	AR_300_	AR_1000_	AR_S_	AR_M_	AR_L_
Based	28.6	49.0	28.3	8.8	34.2	55.8	47.7	47.9	47.9	28.8	56.0	73.2
Based + Soft-NMS	28.7	49.1	28.4	8.9	34.3	55.9	51.9	53.6	54.0	36.4	61.9	76.4
Based + Soft-NMS + VFL	34.3	57.8	33.2	14.4	40.0	61.0	54.9	55.6	55.6	37.9	64.1	77.7

**Table 10:** The Specification of NVIDIA Jetson AGX Xavier.

Parameters	Specifications
CPU	8-core Carmel ARM v8.2 64-bit CPU, 8MB L2 + 4MB L3
GPU	512-core Volta GPU with 64 Tensor Cores 11 TFLOPS (FP16), 22 TOPS (INT8)
Memory	32GB 256-Bit LPDDR4x|136.5GB/s
Toolchain	JetPack 4.4, with: Ubuntu 18.04 CUDA 10.2 cuDNN 8.0

**Table 11:** Model Inference Time Per Frame of NVIDIA Tesla V100.

Model	NN Inference	Post Process	Overall
RetinaNet	18 ms	17 ms	35 ms
RetinaNet-Ours	15 ms	18 ms	34 ms
YOLOv3	13 ms	8 ms	22 ms
YOLOv3-Ours	11 ms	13 ms	26 ms
FCOS	17 ms	10 ms	27 ms
FCOS-Ours	16 ms	10 ms	27 ms
FoveaBox	15 ms	10 ms	26 ms
FoveaBox-Ours	15 ms	10 ms	26 ms
RepPoints	28 ms	4 ms	33 ms
RepPoints-Ours	23 ms	8 ms	32 ms

**Table 12:** Model Inference Time Per Frame of NVIDIA Jetson AGX Xavier.

Model	NN Inference	Post Process	Overall
RetinaNet	66 ms	202 ms	268 ms
RetinaNet-Ours	74 ms	188 ms	264 ms
YOLOv3	52 ms	169 ms	221 ms
YOLOv3-Ours	58 ms	158 ms	218 ms
FCOS	81 ms	152 ms	234 ms
FCOS-Ours	83 ms	153 ms	238 ms
FoveaBox	73 ms	157 ms	231 ms
FoveaBox-Ours	78 ms	156 ms	236 ms
RepPoints	257 ms	20 ms	278 ms
RepPoints-Ours	235 ms	40 ms	277 ms
